# Oxytocin: an unexpected risk for cardiologic and broncho-obstructive effects, and allergic reactions in susceptible delivering women

**DOI:** 10.1186/2049-6958-8-67

**Published:** 2013-10-20

**Authors:** Gennaro Liccardi, Maria Beatrice Bilò, Ciro Mauro, Antonello Salzillo, Amedeo Piccolo, Maria D’Amato, Annabella Liccardi, Gennaro D’Amato

**Affiliations:** 1 Department of Chest Diseases, Division of Pneumology and Allergology. High Speciality “A.Cardarelli” Hospital, Naples, Italy; 2 Allergy Unit, Department of Immunology, Allergy and Respiratory Diseases, University Hospital, Ancona, Italy; 3 Division of Cardiology, Cardiac Intensive Care and Hemodynamic. Department of Intensive Care, High Speciality “A.Cardarelli” Hospital, Naples, Italy; 4 Department of Respiratory Disease, University “Federico II” University – AO “Dei Colli”, Naples, Italy

**Keywords:** Anaphylaxis, Bronchial asthma, Delivery, Drug allergy, Heart, Hypersensitivity, Latex allergy, Oxytocin, Oxytocin allergy, Oxytocin and heart, Oxytocin side effects

## Abstract

Oxytocin (Sintocynon) is considered an uncommon cause of severe allergic reactions during delivery. We have recently shown that allergic sensitization to latex might constitute an important predisposing risk factor for anaphylaxis after the first infusion of oxytocin during delivery.

Some oxytocin cardiovascular activities such as lowering blood pressure, negative cardiac inotropy and cronotropy, parasympathetic neuromodulation, vasodilatation etc. can induce significant side effects mimicking cardiac anaphylaxis, and constitute an additional differential diagnostic problem in delivering women with suspected or real allergic background. Finally, some *ex vivo* models have shown that oxytocin, under pro-inflammatory cytokines stimulation, such as those occurring in asthma, may induce contraction of smooth muscle and airway narrowing.

This background suggests that allergic sensitization to latex allergens constitutes a significant but underestimated risk factor for triggering severe systemic reactions after the infusion of oxytocin and, consequently, there is a need of particular attention in managing delivering women suffering from latex allergy and bronchial asthma. An accurate anamnestic, clinical and diagnostic evaluation, latex-free anesthesiological setting, use of oxytocin-alternative agents and, if necessary, a drug premedication are likely to reduce the risk of anaphylactic/broncho-obstructive reactions in these women.

## Background and Main text

Oxytocin (Sintocynon) is usually considered an uncommon cause of severe allergic reactions during delivery. Very few documented reports on anaphylactic/anaphylactoid reactions as well as severe airway obstruction have been published [1-6]. However, some experimental data highlight the possibility that the risk of developing severe systemic reactions after the infusion of oxytocin during delivery could be higher than expected in some allergic women. In this context, allergic sensitization to latex, the second most frequently incriminated substance inducing anaphylaxis during anesthesia [7], which is also a relatively common condition in female sex [8], might represent an important predisposing risk factor. It is important to outline that hypersensitivity reactions in anesthesia setting are significantly higher in adult women than in men [9]; moreover, Draisci et al. [10] reported a higher prevalence of latex sensitization in the obstetric population than in non-pregnant subjects undergoing gynaecologic surgery.

Ogata and Minami [11] demonstrated homology in the protein sequence of oxytocin and latex allergens Hev b 7.01 and Hev b 7.02 (patatin). These authors suggested that, in their patient sensitized to patatin, subsequent administration of oxytocin could facilitate the antigen recognition, resulting in an anaphylactic response to latex.

We have recently described two life-threatening anaphylactic reactions with onset a few minutes after the infusion of oxytocin in two women sensitized to latex allergens [12]. Both reactions occurred during caesarian section under spinal anaesthesia in the delivery room of our Hospital.

Diagnostic procedures confirmed an IgE - mediated allergic response to both latex and oxytocin, no allergic response was found to other agents used before or during caesarian section such as local antiseptics, proton-pump inhibitors, antihypertensive drugs, low molecular weight heparin (enoxaparin sodium), human albumin.

In view of the few data available in literature, we believe that this topic is underestimated because such adverse events might be easily attributed to latex allergy or to usual “side effects” or alternative/unknown causes. Moreover, this risk could be underestimated if we consider that female sex shows higher prevalence of both hypersensitivity reactions in the anesthesia setting and latex allergy than men.

Among the possible “side effects”, it is important to outline the role of oxytocin on heart [13].

It has been shown that both oxytocin and its receptors are found in the heart and large vessels [14], where accumulating evidence demonstrates cardio protective effects such as natriuresis, altered insulin liberation and anti-diabetic actions, antioxidant actions, inhibition of inflammation, stimulation of endothelial markers in mesenchymal cells and stem cells [15-18].

**Figure 1:**
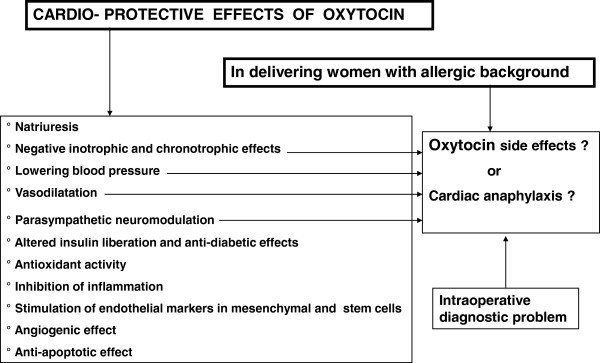
Cardio protection and anaphylactic-like effects of oxytocin.

However, several other cardiovascular activities such as lowering blood pressure, negative cardiac inotropy and cronotropy, parasympathetic neuromodulation, vasodilatation etc. which may be beneficial in some clinical conditions, can induce significant side effects during delivery [13]. These side effects mimicking cardiac anaphylaxis represent an additional differential diagnostic problem in delivering women with suspected or real allergic background [19] (Figure 1). In this context it is important to outline the necessity of serum tryptase determination to confirm the diagnosis of anaphylaxis.

Oxytocin induces uterine contractions during delivery and milk ejection during lactation through activation of a specific G protein-coupled receptor [20]. The expression of this receptor increases before the onset of labour highlighting uterine muscle sensitivity and promoting myometrial shortening.

Interestingly, it has been shown that the expression of oxytocin receptors plays a role not only in uterine but also in other human tissues such as kidney, ovary, heart, vascular endothelium etc. Labour and inflammation increase the expression of oxytocin receptors in human amnion [21], inflammatory conditions may also increase the production of oxytocin receptors in cultivated primary uterine smooth muscle cells [22].

Recently oxytocin receptors have been found also in human airway smooth muscle [23]. Moreover, Amrani et al. [24] have shown that asthma-related cytokines (IL-13 and TNF alpha) modulate the expression of oxytocin receptors in human airway smooth muscle function suggesting a potential role of inflammation-induced changes in oxytocin receptor signaling in the regulation of airway hyper-responsiveness in asthma. In other words, in this *ex vivo* model, oxytocin, under pro-inflammatory cytokines stimulation, may induce contraction of smooth muscle and airway narrowing suggesting that oxytocin serves as a bronchoconstrictor [24]. As a confirmation of this possibility, a case of exclusive severe airway involvement (bronchospasm and laryngeal stridor) after oxytocin administration has also been reported [25].

Taken together, these data suggest that inflammatory conditions of airways such as those found in asthmatic women might constitute an independent (from anaphylaxis) risk factor for airway obstruction after infusion of oxytocin during delivery. The role of oxytocin receptors could also explain the well known worsening of asthma control in about one-third of pregnant women suffering from asthma [26-28].

**Figure 2:**
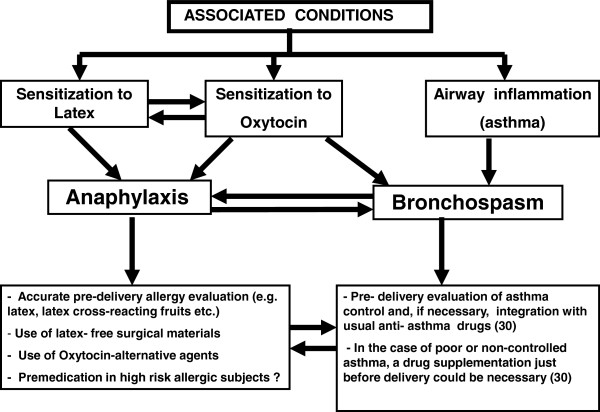
Suggested correlation between latex, oxytocin sensitization and airway inflammatory conditions.

Finally, Gonzalez-Perez et al. have shown that women suffering from severe asthma are at higher risk of anaphylaxis than men [29], as a consequence the risk of developing anaphylaxis, asthma exacerbation or both is likely high in severe asthmatic women (Figure 2).

Since oxytocin causes the alveoli in the breasts to contract causing milk let-down as the milk ejection reflex, there is some controversy over whether or not a woman can be “allergic to breastfeeding”. In fact there are women who have allergy-like symptoms associated with the milk ejection reflex during breastfeeding. These symptoms can include itching, redness, rash or hives on the trunk, arms or legs, anaphylactic reactions as they have been also shown [30]. It has been suggested that these symptoms can also represent adverse reactions to the synthetic forms of oxytocin. Systemic reactions to preservatives contained in preparations of oxytocin has been also described [31].

Although *in vivo* tests with oxytocin have not been standardized, a diluted/undiluted oxytocin solution should be used by skin prick test/intradermal test. In our case report, both patients reacted after the use of skin prick test and, consequently, intradermal test was not necessary [12]. Latex hypersensitivity should be excluded by using *in vivo* (skin prick tests) and *in vitro* (evaluation of specific IgE antibodies by classic or, if possible, micro-array technique) tests.

## Conclusions

In conclusion, our findings suggest a particular attention in managing delivering women suffering from latex allergy and bronchial asthma. An accurate anamnestic, clinical and diagnostic evaluation, latex-free anesthesiological setting, use of oxytocin-alternative agents and, if suffering from asthma, a drug premedication [32] are likely to reduce the risk of anaphylactic or airway-obstructive reactions in these women.

Further *in vitro* studies are necessary to establish the occurrence of an immunological cross-reaction between latex and oxytocin as well as the role of oxytocin and its receptors in heart and airway. Finally, further clinical studies should be designed to a better understanding/management of respiratory and cardiac effects of oxytocin administration.

## Summary statement

Oxytocin may constitute a risk factor for anaphylaxis, bronchial asthma and cardiologic side effects in delivering women.

## Competing interest

All authors declare that they have no conflict of interest and that the study has been carried out without any financial support.
